# The Institutional Worker

**Published:** 1913-03-22

**Authors:** 


THE
### INSTITUTIONAL WORKER
**Being a Special Supplement to "The Hospital"**

### OUR BUREAU OF INFORMATION.

### Rules for Correspondents.

1. Every letter, on whatever subject, must be accompanied by the coupon to be cut from the back cover (inside page) of The Hospital, current issue, and must contain the name and address of the correspondent with pseudonym for publication if desired.
2. Replies by post cannot be given, save under exceptional circumstances at the Editor's discretion.
3. Letters from Approved Homes in reply to special needs published in the Bureau should state terms and full particulars, and be sent prepaid under cover to the Editor of the Bureau with name written across coupon for identification.
4. Proprietors of Homes which have not yet been entered on the List of Approved Homes, but have spare accommodation likely to suit special needs, are invited to write for an application form for registration. The fee for registration, which includes two announcements of the Home in the Bureau and other privileges, is 10s.
5. All communications to be addressed to the Editor of The Hospital, 28 Southampton Street, Strand, London, W.C., and marked "Bureau of Information."

### Training in Children's Hospitals.

You will find a list of children's hospitals and the conditions under which probationers are received for training in "How to Become a Nurse" (2s. 6d., The Scientific Press). We cannot advise you without knowing your age. If you want to enter "for a few months on trial," you can do so in most institutions by paying £1 a week.—Miss V. H.

### Economical Laundry Fittings.

The Editor invites suggestions from correspondents with regard to the economical fitting up of a small laundry for an institution with thirty beds, where hand-power only is available. A list should be given of labour-saving appliances likely to be most useful, with their approximate cost.

### Hostility Towards English Nurses.

We understand that the medical profession in Rouen, with perhaps one or two exceptions, is decidedly hostile to the employment of English nurses. On many occasions it has happened that when a trained nurse has been in attendance on the patient the doctor in charge of the case has barely condescended to notice her. When required by British residents they can always be supplied by the London Hospital. There is evidently no encouragement for British nurses to settle in Rouen under the existing prejudice against a high standard of nursing.—S. T. V.

### Nuns Preferred in Rome.

The demand for English or American nurses varies according to the number of visitors, and is fully met by the Anglo-American Nursing Home, 265 Via Nomentana. Italians greatly prefer the ministration of nuns, and the religious nursing orders are certainly more efficient and better instructed in the duties of the sick-room than formerly. The terms commanded by nurses vary between 5 and 10 lire per day (4s. to 8s.).—W. S. L.

### Prospects in Venice.

If you are spending the spring in Venice, and wish to do visiting nursing there, your right plan would be to call personally on the manager of the principal hotels and leave your card, with terms clearly indicated by the day or hour. Communicate also, of course, with the leading doctors. There is no agency for the supply of British nurses, and when required they are obtained from Florence. It is quite probable you might pay all your expenses if you became known, but it is risky, and unless you can afford the trip irrespective of employment, we cannot recommend your going. Venice gets very hot in May.—Elise.

### Approved Homes.
#### Important Notice.

In consequence of the large amount of correspondence involved in registering Homes, forwarding letters, and communicating with persons in need of accommodation, the Editor is compelled to raise the fees which have hitherto prevailed in this department. After April 1 the registration fee for admission to the Approved List of Homes will be Ten Shillings, to cover also two announcements in the Bureau, and to include the use of the Bureau for forwarding letters for one year from the date of the second announcement of the Home. After the first year the principals of Approved Homes may continue to have their letters forwarded at a charge of Five Shillings a year. All who have received papers, but have not yet sent them in, should forward them not later than Monday, March 31, in order to be admitted to the List at the old rate.

The special needs of any who are sick or in difficulties can be made known in these columns free of charge.

### Home for Invalid Schoolmistress.

You will not find it very easy to procure a really comfortable Home under 12s. 6d., which is the amount you are now paying. As, however, you do not require nursing, it is possible you may be eligible for residential quarters provided for ladies of limited income. Write to Mrs. Lyon, Frithville Homes, 57 and 58 The Grove, Hammersmith, W. You have a strong claim to consideration after your long and honourable service in the teaching profession. The limit of income is not less than £20 and not more than £40, which we gather would suit you. Write also to Mrs. Baron Dickenson, St. Stephen's Vicarage, Lewisham, S.E., and inquire whether you are eligible for the Belmont Home in that locality.—Mrs. A. M. N.

### Feeble-Minded Mother.

You ask whether there is any way to keep in a Home a feeble-minded girl who has three times returned to the workhouse with another illegitimate baby. We regret to inform you that nothing but moral suasion can be used as the

### Out Relief for Children.

We do not consider that the shilling a week which your Board allow for each child is sufficient to provide even bare necessaries in the case of a widow compelled to go out to work. It is not possible to supply adequate food for a growing child at less than 3d. a day, and very good management is needed to do this. It is quite true that among wage-earners with less than 30s. a week the allowance for food for the family, including adults, often sinks to 2d. or even less per head per day, when deductions have been made for rent, insurance, coal, gas, and clothing; but the children who are under the care of the State, even indirectly, are not likely to bring it much credit if slowly starved. Work out for yourself what can be bought for 1¾d. a day in the way of food.—G. B.

### Nurses' Sitting Room.

In furnishing your new nurses' room pay great attention to the choice of chairs. Avoid the cumbersome lounge and rocking-chairs often seen in nurses' quarters, where a few can loll while others groan, and specialise on chairs of one or at most two patterns which shall provide comfort for every occupant of the room. They should afford support for tired arms and a rest for the head, while they should not be so high that a foot-stool is essential to ease. Small low tables, which can be moved about and used for writing or needlework, will be useful; and the aim should be to make the room habitable and handy for occupation rather than to make a smart appearance occasionally for visitors. If you have a cupboard available which can be fitted up with lockers for the nurses' books and work, it will be appreciated, as there is often little time to fetch things from bedrooms at a distance, and many women rest better when their fingers are busy or their minds are amused. — Sister Dorothy.

### Accounts for Small Hospital.

YOU have fallen into a very common confusion through not keeping separate the weekly cost of the food and the bills for stores. It is exceedingly poor economy to buy such things as potatoes, flour, rice, etc., by the pound as required every week, and yet it would be ridiculous to credit the week's accounts with a hundredweight of sugar. If you have inherited this bad system from your predecessor, the sooner you change it the better. Get the "Uniform System of Accounts," Series No. 2, for Cottage Hospitals and Smaller Institutions (The Scientific Press). In reckoning the weekly cost of your household for food you must set down first the weekly bills and then the amount you have used from stores, with the cost of each item. It will facilitate the book-keeping if you reckon out what is the normal consumption for the quarter or the half-year of those articles you keep in stock. Explain to the committee what savings you can effect by buying in bulk, and yourself superintend the weighing and measuring, making a note of everything that is used exactly as if you were buying from a shop.—F. R.

### The Cook's Bouquet.

THE "bouquet garni" is the little bunch of herbs without which French cooks never dream of preparing food. It can be made by walking down any reasonably furnished kitchen garden and plucking a twig here and there. Start with a few sprays of parsley, then add a twig of thyme, of marjoram, of basil, and a leaf or two of celery. Wash these, and fold among them a pepper pod, with a little mace or a clove of garlic, unless the latter is disliked. Other ingredients, such as a small stick of cinnamon or a leaf of sage or of other herbs, can be added. Tie it round, and slide it into the pot or baking dish, the contents of which it is desired to flavour, about half an hour before it is ready for table. It should be removed before the dish is sent to table. A little practice with this simple mode of flavouring will help you not to overdo any of the ingredients so as to drown the combined effect.—NOVICE.

### Bad Habits in the Kitchen.

THE custom to which you refer of blowing the grease off beef-tea has nothing to recommend it, and should not be tolerated in any cleanly kitchen. But you will not be able to stop it unless you supply an easy substitute for this time-honoured law stands. She is at liberty to leave the workhouse with her baby when she is strong enough to do so, and the result is easy to divine. Our experience has been that rescue workers will not undertake cases of this description on account of being overwhelmed with applications of a more hopeful nature. The only hope is in the promised legislation.—GUARDIAN.

### Workhouse Training.

UNTIL you get a well-trained superintendent nurse it is worse than useless to endeavour to attempt training, even of a preliminary nature, to probationers in your sick wards. As the medical officer is non-resident and is strongly opposed to your scheme, we advise you not to bring it forward at present. When changes occur you may introduce your motion; but you should be prepared with a well-thought-out scheme and get the support of other members of the Board. We cannot recommend you to invite applications from girls of eighteen for this work. As matters stand in your institution they might be spoilt for getting further training, and would only worry the other workers.—MISS L.

### Employment for Disfigured Worker.

WE are sorry to hear that your engagement has fallen through, especially as you were so happy and well in the place we sent you to. We will gladly put you in communication with any Home in the country which may offer to give you work at a low salary. Perhaps it is wiser not to go among children. There is plenty of work among chronic and infirm patients, where your help would be valued.—EDWARDES.

⁂ A New Edition of the Leaflet relating to THE HOSPITAL Bureau of Information is now ready and will be forwarded free of charge on application.

---

### Institution Facts and Figures.

### Question for April.

AT the request of a prominent hospital official we again set a question dealing with the laundry and hope we may receive good replies from different types of institutions.

Stating the number of beds in your institution, and the class of patients received, give the following particulars :—

(*a*) The number of garments washed during any four weeks in the winter months, with the price per unit, and the manner in which the cost was arrived at. In addition to wages, fuel, light and power, interest on capital should be taken into account.

(*b*) State the quantity, description and cost of soap used in laundry for the month. If curd and oil soap be used, the amount of alkali should also be stated. State also the quantity of starch and blue.

---

The figures must be the result of actual observation and computation, not estimates. The best answers will be published, and for each contribution considered by the Editor to be of sufficient interest for publication payment of FIVE SHILLINGS will be made.

RULES.

The following rules must be observed by all those answering the Housekeeping Questions :—

1. Answers must be written on one side of the paper. They must bear the name and address of the sender and be accompanied by coupon to be cut from the back cover (inside page) of the current issue of THE HOSPITAL. A pseudonym must be chosen if the name is not to be published.

2. Answers must be addressed to the Editor of THE HOSPITAL, 28-29 Southampton Street, Strand, London, W.C.; they must be marked in the left-hand corner "Facts and Figures," and must be received before the end of the month.

abuse. When skimming has reduced the fat on the surface to tiny dots, the beef-tea or soup may be quickly cleared by letting a clean sheet of blotting-paper just touch the surface. Save the odds and ends of blotting-paper trimmed off when filling blotters and send them down to the cook for this purpose.—SALOME.

### To Make Baking Powder.

THE ingredients are half a pound of tartaric acid and three-quarters of a pound of carbonate of soda. These must be very finely ground, and they should be mixed very thoroughly with three-quarters of a pound of ground rice. Use a heaped teaspoonful with each pound of flour. The cost works out at about 10d. per lb. There is not very much object in making your own mixture, and unless you are careful about mixing, the result will not be satisfactory.—ELLA.

### Appetising Relish.

WHY not serve bread-sauce with the dish of lentils which are found so uninteresting? Strongly flavoured with onion, it makes a very good accompaniment to many dishes besides roast chicken. Allow four ounces of bread-crumbs to a pint of milk. Slice up two or three onions and add a few peppercorns. Boil for ten minutes, stir in a little butter, and serve round the lentils.—S. P. S.

### Nursing Licences in Bohemia.

WE regret to inform you that no foreign nurses are admitted to practise in Bohemia. A licence can only be obtained from the K.R. Allgemeines Krankenhaus, in Prague, where all nurses are obliged to work for one year before obtaining a licence to practise from the head of this Institute. It appears that foreign nurses are not admitted, but we conclude this is merely because more applications are received than can be entertained from Bohemian women anxious to be trained.—DENYS.

### The Editor's Letter-Box.

### Communications have been received and attended to from :—

A. W.; Miss G., Cholsey; Miss L. M.; Miss P.; Miss A. D.; Mr. Th. B.; Miss G., Hale End; H. S.

J. R. M. (145A).—Very glad to know that your patient is so comfortably settled in one of our Approved Homes.

MISS M. F.—Every effort has been made, without success, to trace the missing letter, and we can only conclude that it went astray in the Christmas post. Let us know if we can help you later on.

### Editor's Notices.

**Contributions:**

Contributions should be written, or preferably typed, on one side of the paper only, and all articles sent in are accepted upon the distinct understanding that they are forwarded to THE HOSPITAL only.

The Editor cannot undertake to return MSS. not used, but when a stamped directed envelope is enclosed the MSS. may be returned if a special request is made.

Accepted articles and paragraphs of news will be paid for after publication at the scale rate.

**Address:**

To prevent delay all contributions and letters on editorial business must be addressed exclusively to the Editor, "The Hospital," 29 Southampton Street, Strand, London, W.C. It is important that this regulation shall be strictly observed.

**Correspondence:**

Correspondence on all subjects is invited. The name and address of correspondents must be given as a guarantee of good faith, but not necessarily for publication.

**Special Articles:**

Special articles are invited, and questions, inquiries, and paragraphs upon all matters relating to the work, administration, and management of general, special, mental, fever, cottage and convalescent hospitals, sanatoria, homes, institutions, societies and organisations for the treatment or care of the sick, injured and dependants of all classes. Every matter affecting the interests and welfare of the staff of all grades working in these institutions will receive special consideration.

**Administrative Medicine, Research and Items of News:**

Special terms will be paid for approved articles on subjects in this wide field by experts who have made some section of it their special study and interest. We wish to give prominence to every movement tending to advance accuracy of diagnosis, efficiency in treatment, and the development of modern methods to eradicate disease and promote the welfare of the suffering.

**A Bureau of Information:**

We invite inquiries and applications for information and help in providing for that numerous class of sufferers who require special care or house-room which they cannot obtain in their own homes or secure for themselves. We cannot, however, prescribe, or recommend practitioners.

**Books for Review:**

Publishers are particularly requested to send advance proofs of any new books of importance whenever possible, as the Editor has made arrangements to publish immediate reviews on a new plan.

**Photographs, Plans, Blocks, and Illustrations:**

It is requested that wherever possible MSS. may be accompanied by illustrations in any of the above forms. The name of the sender and of the article to which it belongs should be written on the back of each photograph, drawing, or block for purposes of identification.

**Local Papers:**

Newspapers containing reports or news paragraphs should be marked and addressed to the Sub-Editor.

**The Coupon System:**

A coupon will be found at the bottom of the third inside page of the cover each week. This coupon must be attached to every question or inquiry to which an answer is desired in THE HOSPITAL.

### No Longer in Use.

DOUBTLESS you possess articles that are no longer in use but still in a serviceable condition. Why not turn these into money by disposing of them through our Private Sale and Exchange Columns? This section is reserved exclusively for bargains between our Readers, and privacy in all transactions is assured by the employment of box numbers, *all replies being forwarded through* THE HOSPITAL *Offices.*

Moreover, by utilising our "Deposit System" Readers can with perfect safety forward goods on approval to any part of the country.

Announcements in this section cost 1s. for 14 words and 6d. for 7 words or part of 7 words beyond.

If you have something to sell—some article which is not in use—do not let it rust or rot, but give some other Reader an opportunity of securing it through the medium of THE HOSPITAL Private Sale and Exchange Columns.

Sale and Exchange Announcements, 14 words ... ... 1s.

| | | |
|---|---|---|
| | | |
| | | |
| | | |
| | | |
| | | |
| | | |
| | | |

### Manager's Notices.

**Letters relating to the publication, sale, and advertising departments of "The Hospital" must be addressed to The Manager, "The Hospital," The Hospital Building, 28 and 29 Southampton Street, London, W.C.**

**Subscriptions** may begin at any time, and are payable in advance. Cheques and Post-Office Orders should be crossed London County and Westminster Bank, Covent Garden Branch, and made payable to the Manager of THE HOSPITAL.

RATES OF SUBSCRIPTION (including Postage).

| *United Kingdom.* | | *Foreign and Colonial.* | |
|---|---|---|---|
| Three Months ... | ... 2s. 0d. | Three Months ... | ... 2s. 6d. |
| Six Months ... | ... 4s. 0d. | Six Months ... | ... 5s. 0d. |
| Twelve Months ... | ... 6s. 6d | Twelve Months ... | ... 8s. 8d. |

**Advertisements:**

To ensure insertion, all advertisements for THE HOSPITAL must reach the Manager not later than Wednesday morning in each week. For scale of charges see pages iii and 4.

**Remittances:**

**It is especially requested that remittances be made by Cheque or Postal Order, and in halfpenny stamps only when the amount is under sixpence.**

### Books on Hospitals and Institutions.

By SIR HENRY BURDETT, K.C.B., K.C.V.O.

### HOSPITALS & ASYLUMS OF THE WORLD.

In Four Volumes, with Portfolio of Plans. Complete, £12 12s.

Their Origin, History, Construction, Administration, Management and Legislation; with Plans of the chief Medical Institutions, accurately drawn to a uniform scale.

Vols. I. and II. (only).—Asylums and Asylum Construction, £6 15s.
Vols. III. and IV.—Hospitals and Hospital Construction – with Portfolio of Plans, £9.
The Portfolio of Plans (20in. by 14in.), separately, price £4 14s. 6d. Carriage extra.

IMPORTANT.—AS ONLY A FEW COMPLETE COPIES OF THE WORK REMAIN UNSOLD, Secretaries and Institution Librarians are recommended to take immediate steps to procure this Encyclopædia Britannica of Hospital and Institutional affairs.

### BURDETT'S HOSPITALS & CHARITIES.

The Year Book of Philanthropy, and Hospital Annual.

"The ideal of what a work of reference ought to be."—*The Times.*

Published Annually, over 1000 pages. 10s. 6d. net; 10s. 11d. post free.

This Annual is now in its Twenty-fourth Year, and the complete series form a Library of Hospital Facts and Figures absolutely unobtainable from any other source. Everyone associated with or interested in our Hospitals and Charities should not only obtain the new edition, but all previous issues, since they are a valuable history of the Hospital movement throughout the English-speaking world.

### COTTAGE HOSPITALS, GENERAL, FEVER AND CONVALESCENT.

Their Progress, Management and Work in Great Britain, Ireland and the United States.

Third Edition, profusely illustrated, with nearly 50 Plans, Diagrams, etc. Cloth gilt. 10s. 6d. net.

A New and Abridged Edition of this book is now in the press and will be published shortly.

### THE UNIFORM SYSTEM OF ACCOUNTS.

For Hospitals and all Classes of Public Institutions.

With Special Forms of Accounts and Specimen Rulings of a complete set of books.
New Edition. In the Press.

### ACCOUNT BOOKS FOR INSTITUTIONS.

Designed in accordance with the Uniform System.

(Full description of Books and Prices on application.)

### MEDICAL ATTENDANCE OF LONDONERS.

An Economical System of Medical Relief freed from existing abuses and adequate to protect all classes.

In paper cover. 1s. post free.

### HOW TO BECOME A NURSE.

The Nursing Profession. How and Where to Train.

A Complete Guide to Training for the calling of a Nurse, with particulars of Nurse Training Schools in the United Kingdom and Abroad.
Price 2s. net. By Post, 2s. 4d.

"The advice to women desirous of becoming Nurses is simple, sound and discreet."—*Lancet.*

Of all Booksellers, or of

**The Scientific Press, Limited,** 28/29 SOUTHAMPTON STREET, STRAND. LONDON, W.C.

